# All-cause and cause-specific mortality by spirometric pattern and sex – a population-based cohort study

**DOI:** 10.1177/17534666241232768

**Published:** 2024-03-11

**Authors:** Helena Backman, Sami Sawalha, Ulf Nilsson, Linnea Hedman, Caroline Stridsman, Lowie E. G. W. Vanfleteren, Bright I. Nwaru, Nikolai Stenfors, Eva Rönmark, Anne Lindberg

**Affiliations:** Section of Sustainable Health/The OLIN Unit, Department of Public Health and Clinical Medicine, Umeå University, Umeå, Sweden; Department of Public Health and Clinical Medicine, Umeå University, Umeå, Sweden; Department of Public Health and Clinical Medicine, Umeå University, Umeå, Sweden; Section of Sustainable Health/The OLIN Unit, Department of Public Health and Clinical Medicine, Umeå University, Umeå, Sweden; Department of Public Health and Clinical Medicine, Umeå University, Umeå, Sweden; COPD Center, Sahlgrenska University Hospital, Institute of Medicine, University of Gothenburg, Gothenburg, Sweden; Krefting Research Center, Institution of Medicine, University of Gothenburg, Gothenburg, Sweden; Wallenberg Centre for Molecular and Translational Medicine, University of Gothenburg, Gothenburg, Sweden; Department of Public Health and Clinical Medicine, Umeå University, Umeå, Sweden; Section of Sustainable Health/The OLIN Unit, Department of Public Health and Clinical Medicine, Umeå University, Umeå, Sweden; Department of Public Health and Clinical Medicine, Umeå University, Umeå, Sweden

**Keywords:** epidemiology, chronic airway obstruction, restrictive spirometric pattern, mortality, cause of death

## Abstract

**Background:**

Chronic airway obstruction (CAO) and restrictive spirometry pattern (RSP) are associated with mortality, but sex-specific patterns of all-cause and specific causes of death have hardly been evaluated.

**Objectives:**

To study the possible sex-dependent differences of all-cause mortality and patterns of cause-specific mortality among men and women with CAO and RSP, respectively, to that of normal lung function (NLF).

**Design:**

Population-based prospective cohort study.

**Methods:**

Individuals with CAO [FEV_1_/vital capacity (VC) < 0.70], RSP [FEV_1_/VC ⩾ 0.70 and forced vital capacity (FVC) < 80% predicted] and NLF (FEV_1_/VC ⩾ 0.70 and FVC ⩾ 80% predicted) were identified within the Obstructive Lung Disease in Northern Sweden (OLIN) studies in 2002–2004. Mortality data were collected through April 2016, totally covering 19,000 patient-years. Cox regression and Fine–Gray regression accounting for competing risks were utilized to estimate hazard ratios (HRs) with 95% confidence intervals (CIs) adjusted for age, body mass index, sex, smoking habits and pack-years.

**Results:**

The adjusted hazard for all-cause mortality was higher in CAO and RSP than in NLF (HR, 95% CI; 1.69, 1.31–2.02 and 1.24, 1.06–1.71), and the higher hazards were driven by males. CAO had a higher hazard of respiratory and cardiovascular death than NLF (2.68, 1.05–6.82 and 1.40, 1.04–1.90). The hazard of respiratory death was significant in women (3.41, 1.05–11.07) while the hazard of cardiovascular death was significant in men (1.49, 1.01–2.22). In RSP, the higher hazard for respiratory death remained after adjustment (2.68, 1.05–6.82) but not for cardiovascular death (1.11, 0.74–1.66), with a similar pattern in both sexes.

**Conclusion:**

The higher hazard for all-cause mortality in CAO and RSP than in NLF was male driven. CAO was associated with respiratory death in women and cardiovascular death in men, while RSP is associated with respiratory death, similarly in both sexes.

## Plain language summary

All-cause and cause specific mortality in relation to different lung function patterns and sex; normal, obstructive and restricted lung function

Chronic airway obstruction and restrictive spirometry pattern are associated with mortality, but sex specific patterns have hardly been evaluated.

**Aim:** To study possible sex-dependent differences of all-cause and cause-specific mortality among men and women with chronic airway obstruction and restrictive spirometry pattern, respectively, compared to that of normal lung function.

**Methods:** Individuals with chronic airway obstruction, restrictive spirometry pattern and normal lung function were identified within the Obstructive Lung Disease in Northern Sweden (OLIN) studies in 2002-04. Mortality data were collected through April 2016, totally covering 19,000 patient-years of observation time. We analyzed the Hazard Ratios for all-cause and cause-specific death comparing chronic airway obstruction and restrictive spirometry pattern to that of normal lung function, adjusting for age, body mass index, sex, smoking habits and pack-years. Similar analyses were conducted separately for men and women.

**Results:** The hazard for all-cause mortality was higher in both chronic airway obstruction and restrictive spirometry pattern than in normal lung function and, the higher hazards were male-driven. In chronic airway obstruction the hazard of respiratory and cardiovascular deaths higher than in those with normal lung function. The increased hazard of respiratory death was significant in women while the increased hazard of cardiovascular death was significant in men. In restrictive spirometry pattern, the higher hazard for respiratory but not cardiovascular death persisted after adjustment, similarly in both sexes.

**Conclusions:** The higher hazard for all-cause mortality in chronic airway obstruction and restrictive spirometry pattern than in normal lung function was male-driven. Chronic airway obstruction associated with respiratory death in women and cardiovascular death in men, while restrictive pattern associated with respiratory death, similarly in both sexes.

## Introduction

Chronic obstructive pulmonary disease (COPD) is the most common cause of death worldwide after cardiovascular diseases^1^; however, patients commonly die with COPD and not from COPD.^2,3^ Indeed, increased mortality was seen among patients with chronic respiratory disease in a UK primary care cohort but among them, only 30% of deaths could be attributed to respiratory causes.^4^ Similarly, in a Danish population-based cohort, ischaemic heart disease was the major cause of death among those with obstructive lung disease.^5^ Still, other diseases than COPD may be related to chronic airway obstruction and obstructive lung disease.

Restrictive spirometric pattern (RSP) is about as common as obstructive lung function impairment^6,7^ but the underlying reasons for RSP are heterogeneous and may reflect different disorders, from idiopathic pulmonary fibrosis to thoracic deformities and obesity.^8^ It has been recognized that pulmonary restriction is associated with an increased burden of respiratory symptoms and also represents an independent risk factor for mortality^9–11^ and cardiovascular death.^10,11^ However, specific causes of death remain a rather unexplored topic in this group.

Population-based studies including high-quality spirometry data with post-bronchodilator values for the classification of obstructive lung function and long-term cause-specific mortality data are scarce. Such robust epidemiological analyses are unaffected by the often symptom-driven diagnosis in patient cohorts recruited from healthcare. Thus, a population-based study may contribute to a better understanding of the association between lung function patterns and cause-specific mortality. Importantly, increased knowledge may contribute to the early identification of at-risk patients, providing an opportunity to initiate preventive measures. We have, in a previous publication, confirmed that chronic airway obstruction (CAO) and RSP are associated with similarly increased mortality when compared to normal lung function (NLF) and that an increased risk for respiratory death is the major driver of the increased mortality in both CAO and RSP.^12^

In this paper, the aim was to study possible sex-dependent differences of all-cause mortality and patterns of cause-specific mortality among men and women with chronic airway obstruction and RSP, respectively, in comparison to individuals with NLF, in a long-term follow-up of a population-based cohort.

## Materials and methods

### Study design and study population

The Obstructive Lung Disease in Northern Sweden (OLIN) COPD study has previously been described in detail.^13^ In total, 1986 individuals were included (ages 26–84 years), all 993 individuals with pre-bronchodilator airflow obstruction (FEV_1_/vital capacity (VC) < 0.70) identified after examinations of population-based cohorts, together with 993 age- and sex-matched non-obstructive referents. In this study, cross-sectional data from recruitment, that is, date of examination during 2002–2004, were linked to data on mortality and causes of death through April 2016 obtained from the Swedish National Board of Health and Welfare’s National Cause of Death Register. The reporting of this study conforms to the Strengthening the Reporting of Observational Studies in Epidemiology statement.^14^

### Definitions

Smoking habits were classified into never-smokers, former smokers (had quit smoking since at least 1 year) and current smokers based on past and current use of cigarettes, pipes, cigarillos, etc. Former and current smokers were also grouped as ever-smokers. Pack-years of smoking was calculated among former and current smokers as the number of cigarettes smoked per day/20 × the number of years of smoking. Any respiratory symptoms: the presence of at least any one of the following respiratory symptoms during the last 12 months: longstanding cough, productive cough (cough with phlegm most days during at least 3 months), recurrent wheeze or modified Medical Research Council (mMRC)^15^ dyspnea score ⩾2. Data were collected by a structured interview following a validated questionnaire.^16–18^ Height and weight were measured prior to spirometry. Body mass index (BMI) was calculated as (weight/height^2^) and divided into underweight (<18.5), normal weight (18.5–24.9), overweight (25.0–29.9) and obesity (>30).^19^

### Spirometry and spirometric groups

Spirometry was performed according to the American Thoracic Society’s guidelines^20^ using dry volume spirometers, the Mijnhardt Vicatest 5. VC was defined as the highest of forced vital capacity (FVC) and slow vital capacity (SVC). Reversibility testing was performed with 0.8 mg salbutamol if FEV_1_/VC < 0.70 or FEV_1_ < 80% of predicted. Out of *n* = 993 with pre-bronchodilator airway obstruction, *n* = 736 had CAO, post-bronchodilator FEV_1_/VC < 0.70, corresponding to the spirometric criteria for COPD according to GOLD.^21^ Severity of CAO was divided into *GOLD 1–4*^21^ based on FEV_1_ % of predicted, and included *n* = 272 GOLD 1, *n* = 394 GOLD 2 and *n* = 70 GOLD 3–4. The non-obstructive individuals (FEV_1_/VC ⩾ 0.70, *n* = 993) were divided into NLF (*n* = 742); FEV_1_/VC ⩾ 0.70 and FVC ⩾ 80% of predicted, and RSP (*n* = 251); FEV_1_/VC ⩾ 0.70 and FVC < 80% of predicted. The Swedish OLIN reference values for spirometry were used.^22^

### Causes of death

The tenth version of the International Classification of Diseases (ICD-10)^23^ coding was used for the classification of underlying causes of death. Causes of death are presented in five main groups: Respiratory diseases (J00–J99), cardiovascular diseases (CVD) (I00–I99) and cancer (C00–C97, D00–D48), while the ICD-10 codes F01–09, G30, R549 were grouped under the common term Dementia, and the remaining ICD codes as Others. The main groups are presented in Supplemental Table E1 together with the subgroups relevant for this study.

### Statistical analysis

The Statistical Package for the Social Sciences software version 25, Armonk, NY: IBM Corp and R version 3.6.2, R Core Team was used for statistical analyses. Descriptive statistics are presented as numbers, percentages (%) or means with standard deviations (SD). A *p* value < 0.05 was considered statistically significant. Kaplan–Meier curves illustrate survival from all-cause mortality by spirometric groups. To further assess the association of spirometric groups with all-cause mortality, Cox proportional hazard models were used to calculate hazard ratios (HRs) and 95% confidence intervals (CIs), using left truncation and age as the underlying timescale, and including sex, BMI categories (as only 16 individuals with underweight, they were included in the category normal weight), smoking habits and pack-years as covariates. The proportional hazards assumption was checked based on Schoenfeld residuals. Fine–Gray^24^ competing risk models were used to assess the association of spirometric groups with cause-specific mortality grouped into respiratory, cardiovascular, cancer, dementia and others, adjusted for the same variables as the Cox model. As per previous evidence, our interpretation of the estimated sub-distribution HR from Fine–Gray models relied on the significance and direction of the estimates rather than on their exact magnitude.^25^ The regression analyses were conducted in the total study population and stratified for sex.

## Results

### Clinical characteristics and all-cause mortality

**Figure 1.**
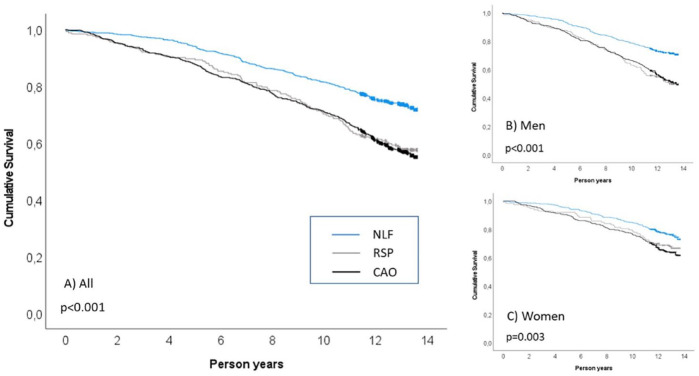
Cumulative survival from all-cause mortality illustrated by Kaplan–Meier curves for individuals with NLF (blue colour), RSP (grey colour) and CAO (black colour). (a) Total study population, (b) among men and (c) among women. Vertical markers on the survival curves represent censored observations. *p* Values from log-rank test. CAO, chronic airway obstruction; NLF, normal lung function; RSP, restrictive spirometric pattern.

In total, there were 607 deaths during the mean follow-up of 10.9 (SD 3.4) years, covering 18,815 person-years (distribution by NLF, RSP and CAO: 8447, 2661 and 7708 person-years, respectively). Clinical characteristics and cumulative mortality by spirometric group and sex are presented in Table 1. Survival from all-cause mortality, illustrated by Kaplan–Meier curves, was on a similar level and worse for those with RSP and CAO than for those with NLF in both women and men (Figure 1), supported also by estimated deaths/1000 person-years (Table 1). Throughout the analysis, the crude all-cause mortality was higher in men than women.

**Table 1.** Clinical characteristics of the population at baseline (at recruitment in 2002–2004) and cumulative all-cause mortality, comparing spirometric groups, by sex.

Variables	Men				Women			
	NLF	RSP	CAO	*p* Value	NLF	RSP	CAO	*p* Value
	*n* = 404	*n* = 138	*n* = 403		*n* = 338	*n* = 113	*n* = 333	
Age, mean (±SD)	62.8 (11.2)	68.0 (10.7)	65.5 (10.7)	**<0.001**	64.3 (11.5)	67.1 (10.3)	65.4 (11.3)	0.062
Interval (min–max)	27–84	36–84	26–84		29–84	33–84	32–84	
BMI mean (±SD)	26.4 (3.3)	27.0 (3.6)	25.9 (3.5)	**0.006**	26.4 (4.1)	27.1 (4.8)	25.5 (4.3)	**<0.001**
Interval (min–max)	20–41	19–38	17–39		17–43	18–47	16–46	
BMI categories								
Underweight	0	0	4 (1.0)		4 (1.2)	1 (0.9)	7(2.1)	
Normal weight	142 (35.1)	44 (31.9)	164 (40.7)		134 (39.6)	44 (38.9)	166 (49.8)	
Overweight	206 (51.0)	69 (50.0)	189 (46.9)		148 (43.8)	41 (36.3)	113 (33.9)	
Obesity	56 (13.9)	25 (18.1)	46 (11.4)	0.053	52 (15.4)	27 (23.9)	47 (14.1)	**0.016**
Smoking habits								
Never smoker	154 (38.1)	56 (40.6)	68 (16.9)		191 (56.5)	74 (65.5)	98 (29.4)	
Former smoker	200 (49.5)	73 (52.9)	189 (46.9)		91 (26.9)	28 (24.8)	102 (30.6)	
Current smoker	50 (12.4)	9 (6.5)	146 (36.2)	**<0.001**	56 (16.6)	11 (9.7)	133 (39.9)	**<0.001**
Pack-years^a^ (±SD)	14.5 (13.5)	12.0 (12.8)	22.6 (17.8)	**<0.001**	11.9 (9.5)	12.2 (14.1)	19.7 (12.8)	**<0.001**
Any respiratory symptoms^b^	154 (38.2)	71 (51.4)	302 (74.9)	**<0.001**	149 (44.1)	58 (51.3)	249 (74.8)	**<0.001**
Productive cough	92 (22.8)	47 (35.9)	205 (50.9)	**<0.001**	64 (18.9)	30 (27.0)	133 (39.9)	**<0.001**
Any exacerbations^c^	22 (5.4)	14 (10.1)	84 (20.8)	**<0.001**	31 (9.2)	22 (19.5)	76 (22.8)	**<0.001**
Cardiovascular diseases^d^	84 (20.8)	44 (31.9)	128 (31.8)	**<0.001**	61 (18.0)	19 (16.8)	64 (19.2)	0.833
Cumulative mortality	113 (28.0)	67 (48.6)	191 (47.4)	**<0.001**	80 (23.7)	37 (32.7)	119 (35.7)	**0.002**
Deaths per 1000 person-years	25	48	47	–	18	29	33	–

Data presented as *n* (%) unless otherwise stated. Estimates with *p* values < 0.05 are bolded. Additionally, deaths/1000 person-years are presented.

a Calculated among ex-smokers and current smokers.

b At least one of the following during the last 12 months: longstanding cough, productive cough (cough with phlegm most days during at least 3 months), recurrent wheeze or mMRC score ⩾2.

c Contacted health care due to respiratory complaints during the last 12 months.

d Heart disease (including angina pectoris, myocardial infarction, CABG and/or PCI, claudication or cerebrovascular disease).

CABG, coronary artery bypass grafting; CAO, chronic airflow obstruction; NLF, normal lung function; PCI, percutaneous coronary intervention; RSP, restrictive spirometry pattern; SD, standard deviation.

### Cause-specific mortality

**Figure 2.**
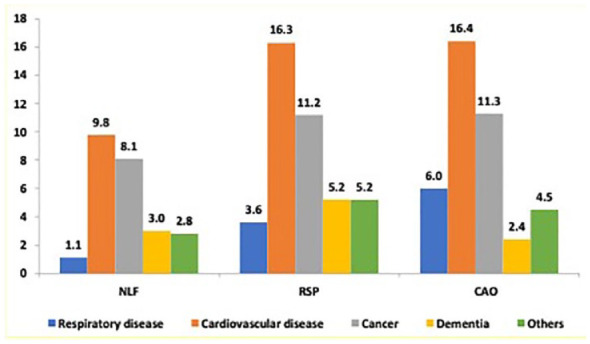
Cumulative mortality (%) due to respiratory diseases (ICD-10 J00–J99), cardiovascular diseases (I00–I99), cancer (C00–C97 and D00–D48), dementia (F01–09, G30 and R549) and other causes of death among individuals with NLF, RSP and CAO, respectively. CAO, chronic airway obstruction; ICD-10, tenth version of the International Classification of Diseases; NLF, normal lung function; RSP, restrictive spirometric pattern.

The cumulative cause-specific mortality in the main five groups (respiratory, cardiovascular, cancer, dementia and others) is illustrated by spirometric pattern in Figure 2. The cumulative cause-specific mortality of the main five groups is presented in Table 2 by spirometric pattern and sex, also including details on the subgroups with less frequent causes. Among respiratory deaths, chronic lower respiratory disease was the cause of death for 88.9% in CAO compared to 55.6% in RSP, while lower respiratory tract infection was the cause of death for 8.9% in CAO and 44.4% in RSP. Cardiovascular mortality was the most common cause-specific cause of death in all groups with ischaemic heart disease being the most prevalent, 48.7% in NLF, 56.1% in RSP and 46.0% in CAO. The cumulative mortality due to cancer was similar in all groups but the proportion of intrathoracic cancer (respiratory and intrathoracic organs, mesothelioma) was highest (28.6%) in CAO. Distribution of all specific causes of death following the structure of the ICD-10 codes chapters are presented by sex in Supplemental Table E2.

**Table 2.** Cumulative cause-specific mortality (respiratory diseases, cardiovascular diseases, cancer, dementia and others) among men and women with NLF, RSP and CAO, respectively.

Cause of death	ICD-10 codes	Men			Women		
		NLF	RSP	CAO	NLF	RSP	CAO
		*n* = 404	*n* = 138	*n* = 403	*n* = 338	*n* = 113	*n* = 333
**Respiratory disease**, *n* (%)	**J00–J99**	**5 (1.2)**	**6 (4.3)**	**23 (5.7)**	**3 (0.9)**	**3 (2.7)**	**22 (6.6)**
*Pneumonia, lower respiratory infections*	*J09–J22*	*2*	*4*	*3*	*2*	*0*	*1*
*Chronic lower respiratory diseases*	*J40-47*	*1*	*2*	*19*	*0*	*3*	*21*
*Other respiratory diseases*	*J84*	*2*	*0*	*1*	*1*	*0*	*0*
**Cardiovascular disease**, *n* (%)	**I00–I99**	**47 (11.6)**	**25 (18.1)**	**79 (19.6)**	**29 (8.6)**	**16 (14.2)**	**47 (14.1)**
*Ischaemic heart disease*	*I20–I25*	*22*	*14*	*36*	*15*	*9*	*22*
*Cerebrovascular diseases*	*I60–I69*	*13*	*6*	*21*	*7*	*5*	*10*
*Hypertensive and other diseases of the circulatory system*	*I10–15*	*12*	*5*	*22*	*7*	*2*	*15*
**Cancer**, *n* (%)	**C00–C97, D00–D48**	**35 (8.7)**	**19 (13.8)**	**53 (13.2)**	**24 (7.1)**	**9 (8.0)**	**31 (9.3)**
*Respiratory and intrathoracic organs, mesothelioma*	*C30–C39, C45*,^a^ *D38*^a^	*0*	*2*	*17*	*2*	*0*	*7*
**Dementia**, *n* (%)	**F01–09, G30, R549**	**11 (2.7)**	**8 (5.8)**	**14 (3.5)**	**13 (3.8)**	**5 (4.4)**	**7 (2.1)**
**Other causes of death**, *n* (%)	Other than the above	**15 (3.7)**	**9 (6.5)**	**22 (5.5)**	**11 (3.3)**	**4 (3.5)**	**12 (3.6)**

Within the main groups respiratory, cardiovascular and cancer also subgroups are presented in italics. Data presented as *n* (%) by the main group, and as *n* per diagnoses within the group’s respiratory disease, cardiovascular disease and cancer).

a One individual.

CAO, chronic airway obstruction; ICD-10, tenth version of the International Classification of Diseases; NLF, normal lung function; RSP, restrictive spirometric pattern.

### HRs for all-cause and cause-specific mortality

**Figure 3.**
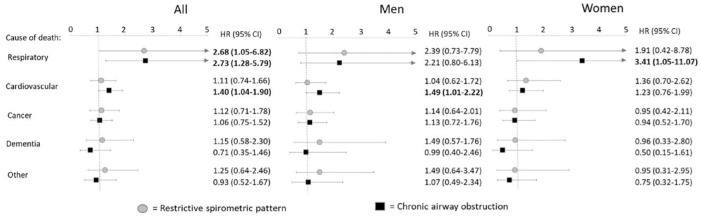
Sub-distribution HRs with 95% CIs for different causes of death from competing risk models adjusted for age, smoking habits, pack years and BMI category. In analyses among all, the models are additionally adjusted for sex. Grey markers represent HR (95% CI) of RSP and black markers represent HR (95% CI) of CAO, both compared to the reference category normal lung function. The results are presented among all (adjusted for sex) and separately for men and women. Bold font indicates *p* < 0.05. CAO, chronic airway obstruction; CI, confidence intervals; HR, hazard ratios; RSP, restrictive spirometric pattern.

The hazard for all-cause mortality was higher in both RSP and CAO when compared with NLF and also when adjusting for age, sex, BMI, smoking habits and pack years (Table 3). This was also the case for respiratory-specific mortality for both RSP (HR: 2.68; 95% CI: 1.05–6.82) and CAO (2.73; 1.28–5.79) and for cardiovascular-specific mortality in CAO only (1.40; 1.04–1.90) (Figure 3).

**Table 3.** Adjusted hazard for all-cause mortality comparing RSP and CAO to those with NLF, expressed as Hazard Ratios (HR) with 95% CIs from Cox regression models.

	All		Men		Women	
	Adjusted^a^		Adjusted^b^		Adjusted^b^	
	HR	(95% CI)	HR	(95% CI)	HR	(95% CI)
NLF	1		1		1	
RSP	**1.28**	**(1.00–1.63)**	1.32	(0.97–1.79)	1.15	(0.77–1.72)
CAO	**1.33**	**(1.10–1.62)**	**1.53**	**(1.19–1.97)**	1.12	(0.83–1.52)

Analyses of the total study population (all) and also stratified for sex.

a Including the covariates sex, BMI categories (normal weight/overweight/obesity), smoking habits (never smoker/former smoker/current smoker) and pack-years while using left truncation and age as the underlying timescale.

b Identical to footnote ‘a’ but without sex-adjustment, estimates with *p* values < 0.05 are bolded.

CAO, chronic airway obstruction; CI, confidence interval; HR, hazard ratio; NLF, normal lung function; RSP, restrictive spirometry pattern.

The analyses stratified for sex revealed that the increased hazard for all-cause mortality in CAO was mainly driven by men (Table 3) and tended to be so also in RSP. The sex-stratified analyses of cause-specific mortality are illustrated in Figure 3. In CAO, the increased HR for respiratory death was significant in women (HR: 3.41; 95% CI: 1.05–11.07) while the hazard for cardiovascular death was significantly elevated in men (1.49; 1.01–2.22). When dividing CAO into severity stages, the increased hazard for death due to respiratory and cardiovascular disease was mainly driven by GOLD stages ⩾2 in general and in both sexes (Supplemental Table E3). In RSP, there were no indications of differences in respiratory-specific mortality between sexes. Nor were there any increased hazards of dying from cancer, dementia or other causes of death, in either sex, when comparing RSP or CAO with NLF.

## Discussion

This population-based cohort study, covering close to 19,000 person-years during a mean of 10.9 years follow-up, showed that reduced lung function is associated with an increased hazard of death and that the association between spirometric pattern (obstructive *versus* restrictive) and cause-specific mortality differed between sexes. Indeed, in CAO, the increased hazard of respiratory mortality was mainly driven by women while the increased hazard of cardiovascular death was driven by men. In RSP, however, the increased hazard for respiratory cause of death was fairly similar in men and women.

The novelty of our study is the opportunity to present underlying causes of death contributing to the increased hazard for all-cause mortality among individuals with RSP and CAO, taking competing risks into account also in analyses stratified for sex. It is known that individuals with chronic airway obstruction or COPD have an increased risk of all-cause mortality compared to others, not only supported by registry-based studies^4,26–28^ but also population-based studies.^5,9,29–31^ Similarly, pulmonary restriction [RSP or Preserved Ratio Impaired Spirometry (PRISm)] was associated with increased all-cause mortality both in patients from clinical centres^32,33^ and population-based studies.^8,9,11^ Our study confirmed an increased hazard for all-cause mortality in CAO as well as RSP when compared with NLF, and the increased hazard in CAO was driven by men and tended to be so also in RSP.

The adjusted hazard of respiratory death among both those with RSP and CAO was substantially higher than in individuals with NLF and, among those with CAO, significantly so among women but not men. The hazard for respiratory death increased by the severity of airflow obstruction, in line with previous studies,^5,31,34,35^ and was highest in GOLD 3–4 in both sexes. However, it appeared that respiratory causes of death had a different pattern among those with CAO and those with RSP. In CAO, respiratory deaths were mainly due to chronic lower respiratory disease, while in RSP, almost every other respiratory death was due to lower respiratory tract infections. We have previously shown that the different comorbidity profiles in men and women were associated with mortality.^36^ However, sex-dependent patterns have rarely been evaluated in the current context, that is, comparing cause-specific mortality by spirometric groups. One explanation for the stronger association between CAO and respiratory mortality in women than men could be an increased female vulnerability to the harmful effects of tobacco smoke.^37^

Cardiovascular disease, and particularly ischaemic heart disease, is the leading cause of death in the world^1^ and is acknowledged as a leading cause of death also among individuals with chronic airway obstruction.^5,35,38,39^ Also clinical pharmaceutical trials including selected patient populations with COPD have provided detailed results on cardiovascular mortality.^40–42^ Even though cardiovascular death was the most common cause of death in all spirometric groups in the current study, it was at a similar and higher level in RSP and CAO than NLF, corresponding to the pattern of reported cardiovascular disease at baseline. In the adjusted models, the increased hazard of cardiovascular death persisted in CAO, significantly so in men but not women, but was lost among those with RSP. The lack of association between RSP and cardiovascular death should, however, be interpreted with caution due to the low number of RSPs. The stronger association between CAO and cardiovascular mortality in men than women may relate to a higher prevalence of cardiovascular disease in men, but potentially also to under-recognition of cardiovascular disease in women.^43^ For comparison, the risk for cardiovascular disease was increased in both those with obstructive and restrictive lung function impairment in the large National Health and Nutrition Examination Survey (NHANES) in the United States.^44^ Other studies have also indicated an increased burden of cardiovascular disease, both assessed as hospital admissions^12^ and mortality^11^ among individuals with pulmonary restriction; however, the referred studies did not include analyses stratified for sex.

In Sweden,^45^ as in most high- and middle-income countries,^46^ cancer is the second leading cause of death, and so it was in the current study. The cumulative cancer mortality was on a similar level in all spirometric groups, and so was the adjusted hazard for cancer death, in both sexes and each of the GOLD stages in CAO. Lung cancer is, by far, the leading cause of cancer deaths worldwide^1,46^ and may be responsible for close to every fifth cancer death. In the current study, almost 30% of the cancer deaths among those with CAO were due to respiratory cancer, compared to only 3.4% in NLF and 7.1% in RSP, not unexpected given the differences in smoking habits between groups. For comparison, in the smoking COPD population included in the Lung Health study, 60% of all deaths were due to cancer, and lung cancer was the most common, encompassing 38% of all deaths.^40^

In addition to the main groups of cause-specific death (respiratory, cardiovascular and cancer), different ICD codes that could be incorporated into the concept of dementia (Alzheimer, dementia, senility, vascular dementia) were common. This corresponds well with WHO’s data where dementia has emerged among the top 10 causes of death in the world.^1^ Even though no differences between spirometric groups or sexes were observed in our study, this is still a topic for future research of clinical importance due to the increasing life expectancy in high- and middle-income countries.

A reduced COPD mortality among men has been observed in the United States since the 1970s and, in parallel, an increased COPD mortality among women.^47^ A similar pattern has later been observed in Sweden where the number of COPD deaths per 100,000 individuals has been higher in women than men since the mid-2000s according to the Swedish National Board of Health and Welfare’s National Cause of Death Register. The changes observed over time in register-based data emphasize the need for future studies to follow potential further emerging differences in specific causes of death among men and women with COPD.

The strength of this study is the long observation time of a large population-based cohort including post-bronchodilator spirometry for the definition of CAO corresponding to the GOLD^21^ spirometric criteria for COPD. Still, we preferred the term CAO to COPD as the definition was based on spirometric findings without clinical assessment of symptoms. In addition, the population-based design and post-bronchodilator spirometry increase the generalizability by minimizing the risk of under-diagnosis^48,49^ and misclassification^50^ of chronic airway obstruction affecting the results of clinical cohort and registry-based studies. Another strength of the current study is that there was no missing data on mortality, as registration of the cause of death is compulsory in Sweden.

The original design of the OLIN COPD study aimed at comparing individuals with and without airway obstruction, which contributed to a low statistical power for RSP. A further limitation is that only baseline spirometry was included and RSP may, in some cases, represent a transitional state^12^ also including development into airway obstruction.^10,29^ Even though the CAO cohort was large, the size of the study population limits the ability to conduct subgroup analyses, and more studies are needed to disentangle the sex-dependent differences indicated by our study. Furthermore, spirometric criteria for airway obstruction are not synonymous with COPD, and there may be other reasons for airway obstruction or concomitant restricted lung function that cannot be distinguished due to the limitations of dynamic spirometry. Moreover, in Sweden as well as in other Nordic countries, death certificates are most often based on clinical diagnosis which may introduce uncertainties when compared to autopsies. Clinical autopsies are rarely performed unless the individual died in an unnatural way or unclear circumstances calling for a forensic autopsy. Nevertheless, death certificates are today the best data available for population-based studies on causes of death.

## Conclusion

Individuals with restrictive (RSP) and obstructive (CAO) spirometric patterns had a male-driven higher all-cause mortality than those with NLF. The higher mortality in CAO was driven by an increased hazard of respiratory death, which was most pronounced among women, but also cardiovascular death, however, most pronounced among men. RSP had an increased hazard for respiratory, but not cardiovascular death, of similar magnitude in both sexes. Future studies are needed to provide further insight into the observed sex-dependent differences in cause-specific death and may contribute to a basis for targeted preventive measures.

## Supplemental Material

sj-doc-2-tar-10.1177_17534666241232768 – Supplemental material for All-cause and cause-specific mortality by spirometric pattern and sex – a population-based cohort study Supplemental material, sj-doc-2-tar-10.1177_17534666241232768 for All-cause and cause-specific mortality by spirometric pattern and sex – a population-based cohort study by Helena Backman, Sami Sawalha, Ulf Nilsson, Linnea Hedman, Caroline Stridsman, Lowie E. G. W. Vanfleteren, Bright I. Nwaru, Nikolai Stenfors, Eva Rönmark and Anne Lindberg in Therapeutic Advances in Respiratory Disease

sj-docx-1-tar-10.1177_17534666241232768 – Supplemental material for All-cause and cause-specific mortality by spirometric pattern and sex – a population-based cohort study Supplemental material, sj-docx-1-tar-10.1177_17534666241232768 for All-cause and cause-specific mortality by spirometric pattern and sex – a population-based cohort study by Helena Backman, Sami Sawalha, Ulf Nilsson, Linnea Hedman, Caroline Stridsman, Lowie E. G. W. Vanfleteren, Bright I. Nwaru, Nikolai Stenfors, Eva Rönmark and Anne Lindberg in Therapeutic Advances in Respiratory Disease

## Declarations

**Ethics approval and consent to participate:** Ethical approvals were given by the Regional Ethics Committee at Umeå University (Dnr 04-045 M and 215/446-31Ö, date of approval 24 February 2004 and 17 May 2016, respectively). Individuals participating in the OLIN COPD study provided written informed consent. The study complies with the declaration of Helsinki.

**Consent for publication:** Not applicable.

**Availability of data and materials:** The dataset used and analysed during the current study is available from the corresponding author on reasonable request and with ethical permission. The used questionnaire in Swedish is also available upon request.
