# Supplementary material for: All-cause and cause-specific mortality by spirometric pattern and sex – a population-based cohort study
Source: Ther Adv Respir Dis. 2024 Mar 11;18:17534666241232768. doi: 10.1177/17534666241232768 (PMC10929033; doi:10.1177/17534666241232768)
Supplement: sj-doc-2-tar-10.1177_17534666241232768 – Supplemental material for All-cause and cause-specific mortality by spirometric pattern and sex – a population-based cohort study [file sj-doc-2-tar-10.1177_17534666241232768.doc]

STROBE Statement—checklist of items that should be included in reports of observational studies

|  | Item No | Recommendation |
| --- | --- | --- |
| **Title and abstract** | 1 | (*a*) Indicate the study’s design with a commonly used term in the title or the abstract  YES, page 1 |
|  |  | (*b*) Provide in the abstract an informative and balanced summary of what was done and what was found  YES, page 2 |
| Introduction | | |
| Background/rationale | 2 | Explain the scientific background and rationale for the investigation being reported  YES, page 4 and 5 |
| Objectives | 3 | State specific objectives, including any prespecified hypotheses  YES, page 4, last paragraph of the section Introduction |
| Methods | | |
| Study design | 4 | Present key elements of study design early in the paper  YES, page 6, first paragraph of the section Material and Methods |
| Setting | 5 | Describe the setting, locations, and relevant dates, including periods of recruitment, exposure, follow-up, and data collection  YES, page 6, first paragraph of the section Material and Methods |
| Participants | 6 | (*a*) *Cohort study*—Give the eligibility criteria, and the sources and methods of selection of participants. Describe methods of follow-up  YES, page 6, first paragraph of the section Material and Methods  *Case-control study*—Give the eligibility criteria, and the sources and methods of case ascertainment and control selection. Give the rationale for the choice of cases and controls  YES, page 6, first paragraph of the section Material and Methods  *Cross-sectional study*—Give the eligibility criteria, and the sources and methods of selection of participants |
|  |  | (*b*) *Cohort study*—For matched studies, give matching criteria and number of exposed and unexposed  *Case-control study*—For matched studies, give matching criteria and the number of controls per case |
| Variables | 7 | Clearly define all outcomes, exposures, predictors, potential confounders, and effect modifiers. Give diagnostic criteria, if applicable  YES, page 6 and 7, relevant data is presented in the section Material and Methods |
| Data sources/ measurement | 8* | For each variable of interest, give sources of data and details of methods of assessment (measurement). Describe comparability of assessment methods if there is more than one group  YES, page 6 and 7, relevant data is presented in the section Material and Methods; structured interview, height, weight, spirometry and data from the National Board of Health and Welfare Cause of Death register. |
| Bias | 9 | Describe any efforts to address potential sources of bias  Page 7 and 8, paragraph “Statistical analysis” in the section Material and Methods  Analyses of hazards ratios for all-cause mortality and cause-specific death were adjusted for age, sex, BMI-categories, smoking habits and pack-years. Corresponding analyses were conducted stratified for sex including the co-variates age, BMI-categories, smoking habits and pack-years. The analyses were conducting taking competing risk into account (Fine - Gray). |
| Study size | 10 | Explain how the study size was arrived at  Page 6, first paragraph in the section Material and Methods.  After re-examinations in 2002-04 of previously examined individuals in four population-based cohorts, all individuals with airway obstruction (FEV_1_/VC) were identified (n=993) together with age- and sex-matched individuals without airway obstruction (n=993); the study population include in total 1986 individuals. |
| Quantitative variables | 11 | Explain how quantitative variables were handled in the analyses. If applicable, describe which groupings were chosen and why  Page 7, paragraph “Statistical analysis”, descriptive statistics, n(%) and mean (SD) values |
| Statistical methods | 12 | (*a*) Describe all statistical methods, including those used to control for confounding  Page 7 and 8, paragraph “Statistical analysis” in the section Material and Methods and also answer to question 9 |
|  |  | (*b*) Describe any methods used to examine subgroups and interactions  Page 8, paragraph “Statistical analysis” in the section Material and Methods, the last sentence present that analyses stratified for sex were included. |
|  |  | (*c*) Explain how missing data were addressed  Page 6. Recruitment to the OLIN COPD study is based data from participation at the re-examinations in 2002-04, thus there are no missing data. It is compulsory to register cause of death in Sweden, thus there were no missing data on mortality or cause of death due to study design. |
|  |  | (*d*) *Cohort study*—If applicable, explain how loss to follow-up was addressed  Please see answer to question 12.c  *Case-control study*—If applicable, explain how matching of cases and controls was addressed  Please see question 10.  *Cross-sectional study*—If applicable, describe analytical methods taking account of sampling strategy  Please see question 10 and 12 regarding cross-sectional data at recruitment. |
|  |  | (*e*) Describe any sensitivity analyses  Not applicable |

Continued on next page

| Results | | |
| --- | --- | --- |
| Participants | 13* | (a) Report numbers of individuals at each stage of study—eg numbers potentially eligible, examined for eligibility, confirmed eligible, included in the study, completing follow-up, and analysed  Page 6 and 7. At recruitment in total 993 individuals with airway obstruction together with age- and sex-matched individuals without airway obstruction; in total n=1986. Spirometric groups are presented under the headline *Spirometyr and spirometric groups* together with information on participating groups and n in each group.  Page 6. Follow-up: all-cause mortality and cause-specific death until April 2016. |
|  |  | (b) Give reasons for non-participation at each stage  Not applicable |
|  |  | (c) Consider use of a flow diagram  We believe it is not needed |
| Descriptive data | 14* | (a) Give characteristics of study participants (eg demographic, clinical, social) and information on exposures and potential confounders  Table 1 |
|  |  | (b) Indicate number of participants with missing data for each variable of interest  Not applicable |
|  |  | (c) *Cohort study*—Summarise follow-up time (eg, average and total amount)  From recruitment (2002-2004) until death through April 2016 |
| Outcome data | 15* | *Cohort study*—Report numbers of outcome events or summary measures over time  All-cause mortality and cause of death; Figure 1 (Kaplan Meier curves), Tables 1 and 2, Online Table E2 |
|  |  | *Case-control study—*Report numbers in each exposure category, or summary measures of exposure  Page 6 and 7, individuals with airway obstruction (case, n=993) and without airway obstruction (controls, n=993). Spirometric groups are presented under the headline *Spirometyr and spirometric groups* together with information on participating groups and n in each group. |
|  |  | *Cross-sectional study—*Report numbers of outcome events or summary measures  Not applicable |
| Main results | 16 | (*a*) Give unadjusted estimates and, if applicable, confounder-adjusted estimates and their precision (eg, 95% confidence interval). Make clear which confounders were adjusted for and why they were included  Page 7 and 8, ‘Statistical analysis’ in the section Material and Methods, Table 3, Online table E3 and Figure 3. |
|  |  | (*b*) Report category boundaries when continuous variables were categorized  Added intervals (min – max) for age and BMI in Table 1. |
|  |  | (*c*) If relevant, consider translating estimates of relative risk into absolute risk for a meaningful time period  Not applicable |
| Other analyses | 17 | Report other analyses done—eg analyses of subgroups and interactions, and sensitivity analyses  Not applicable besides already presented data in text, tables and figures |
| Discussion | | |
| Key results | 18 | Summarise key results with reference to study objectives  Page 11, first paragraph, |
| Limitations | 19 | Discuss limitations of the study, taking into account sources of potential bias or imprecision. Discuss both direction and magnitude of any potential bias  Page 14-15 |
| Interpretation | 20 | Give a cautious overall interpretation of results considering objectives, limitations, multiplicity of analyses, results from similar studies, and other relevant evidence  Pages 11-15, strengths and limitations specifically page 14-15 |
| Generalisability | 21 | Discuss the generalisability (external validity) of the study results  Page 14 |
| Other information | | |
| Funding | 22 | Give the source of funding and the role of the funders for the present study and, if applicable, for the original study on which the present article is based  Page 17 under the sub-headline *Fundeing* |

*Give information separately for cases and controls in case-control studies and, if applicable, for exposed and unexposed groups in cohort and cross-sectional studies.

**Note:** An Explanation and Elaboration article discusses each checklist item and gives methodological background and published examples of transparent reporting. The STROBE checklist is best used in conjunction with this article (freely available on the Web sites of PLoS Medicine at http://www.plosmedicine.org/, Annals of Internal Medicine at http://www.annals.org/, and Epidemiology at http://www.epidem.com/). Information on the STROBE Initiative is available at www.strobe-statement.org.
