# Supplementary material for: All-cause and cause-specific mortality by spirometric pattern and sex – a population-based cohort study
Source: Ther Adv Respir Dis. 2024 Mar 11;18:17534666241232768. doi: 10.1177/17534666241232768 (PMC10929033; doi:10.1177/17534666241232768)
Supplement: sj-docx-1-tar-10.1177_17534666241232768 – Supplemental material for All-cause and cause-specific mortality by spirometric pattern and sex – a population-based cohort study [file sj-docx-1-tar-10.1177_17534666241232768.docx]

**All-cause and cause-specific mortality by spirometric pattern and sex**

**– The OLIN COPD study**

**Online supplement**

Helena Backman^*^, Sami Sawalha^*^, Ulf Nilsson, Linnea Hedman, Caroline Stridsman, Lowie E. G. W. Vanfleteren, Bright I. Nwaru, Nikolai Stenfors, Eva Rönmark, Anne Lindberg

*These two authors have contributed equally to first authorship.

Online Table E1. Cause of death by ICD codes divided into five main groups in bold, together with

the used subgroups in italics.

|  | **ICD-10 codes** |
| --- | --- |
| **Respiratory disease** | **J00-J99** |
| Pneumonia, Lower respiratory infections | J09-J22 |
| Chronic lower respiratory diseases | J40-J47 |
| Other respiratory diseases | J88 |
| **Cardiovascular disease** | **I00-I99** |
| *Ischemic heart disease* | *I20-I25* |
| *Cerebrovascular disease* | *I60-I69* |
| *Hypertensive and other diseases of the circulatory system* | *I10-I15* |
| **Cancer** | **C00-C97, D00-D48** |
| *Respiratory and intrathoracic organs, mesothelioma* | *C30-39, C45, D38* |
| **Dementia** | **F01-09, G30, R549** |
| **Other causes of death** | **Other than the above** |

Online Table E2. Distribution of cumulative mortality by specific underlying causes of death by sex among those with normal lung function (NLF), restrictive spirometry pattern (RSP) and chronic airflow limitation (CAO), respectively, following the structure of the ICD-10 codes chapters.^1^ Data are presented as n (%) within each column.

|  |  |  | **Number of deceased** | | | | | |
| --- | --- | --- | --- | --- | --- | --- | --- | --- |
|  |  |  | **Men** | | | **Women** | | |
|  |  |  | **NLF** | **RSP** | **CAO** | **NLF** | **RSP** | **CAO** |
| Chapter |  | **ICD-10** | n=113 | n=67 | n=191 | n=80 | n=37 | n=119 |
| I | Certain infectious and parasitic diseases | [A00-B99](http://icd.internetmedicin.se/diagnos/A00-B99-Vissa-infektionssjukdomar-och.html) | 2 (1.8) | 0 | 5 (2.6) | 1 (1.3) | 0 | 1 (0.8) |
| II | Neoplasms | [C00-D48](http://icd.internetmedicin.se/diagnos/C00-D48-Tumorer.html) | 36 (31.9) | 19 (28.4) | 53 (27.7) | 24 (30.0) | 9 (24.3) | 31 (26.1) |
| III | Diseases of the blood and blood-forming organs and certain disorders involving the immune mechanism | D50-D89 | 1 (0.9) | 0 | 0 | 0 | 0 | 0 |
| IV | Endocrine, nutritional and metabolic diseases | [E00-E90](http://icd.internetmedicin.se/diagnos/E00-E90-Endokrina-sjukdomar-nutritionsrubbningar-och.html) | 2 (1.8) | 1 (1.5) | 0 | 3 (3.8) | 0 | 2 (1.7) |
| V | Mental and behavioral disorders | [F00-F99](http://icd.internetmedicin.se/diagnos/F00-F99-Psykiska-sjukdomar-och-syndrom-samt.html) | 9 (8.0) | 5 (7.5) | 10 (5.2) | 5 (6.3) | 2 (5.4) | 5 (4.2) |
| VI | Diseases of the nervous system | [G00-G99](http://icd.internetmedicin.se/diagnos/G00-G99-Sjukdomar-i-nervsystemet.html) | 4 (3.5) | 4 (6.0) | 5 (2.6) | 7 (8.8) | 2 (5.4) | 1 (0.8) |
| IX | Diseases of the circulatory system | [I00-I99](http://icd.internetmedicin.se/diagnos/I00-I99-Cirkulationsorganens-sjukdomar.html) | 47 (41.6) | 25 (37.3) | 79 (41.4) | 29 (36.3) | 16 (43.2) | 47 (39.5) |
| X | Diseases of the respiratory system | [J00-J99](http://icd.internetmedicin.se/diagnos/J00-J99-Andningsorganens-sjukdomar.html) | 5 (4.4) | 6 (9.0) | 23 (12.0) | 3 (3.8) | 3 (8.1) | 22 (18.5) |
| XI | Diseases of the digestive system | [K00-K93](http://icd.internetmedicin.se/diagnos/K00-K93-Matsmaltningsorganens-sjukdomar.html) | 2 (1.8) | 2 (3.0) | 5 (2.6) | 0 | 0 | 6 (5.0) |
| XIV | Diseases of the genitourinary system | [N00-N99](http://icd.internetmedicin.se/diagnos/N00-N99-Sjukdomar-i-urin--och-konsorganen.html) | 1 (0.9) | 0 | 1 (0.5) | 1 (1.3) | 1 (2.7) | 0 |
| XVIII | Symptoms, signs and abnormal clinical and laboratory findings, not elsewhere classified | [R00-R99](http://icd.internetmedicin.se/diagnos/R00-R99-Symtom-sjukdomstecken-och-onormala-kliniska.html) | 0 | 1 (1.5) | 0 | 2 (2.5) | 2 (5.4) | 1 (0.8) |
| XX | External causes of morbidity and mortality | [V01-Y98](http://icd.internetmedicin.se/diagnos/V01-Y98-Yttre-orsaker-till-sjukdom-och-dod.html) | 4 (3.5) | 4 (6.0) | 10 (5.2) | 5 (6.3) | 2 (5.4) | 3 (2.5) |

^1^The presented ICD-codes chapters cover underlying cause of death among all deceased during the follow-up of the study.

Online table E3: Risk for all-cause mortality, respiratory, cardiovascular and cancer causes of death among individuals with RSP and CAO by GOLD stage compared to individuals with NLF. All-cause mortality is analyzed in a Cox regression model and cause-specific mortality in a Fine and Gray regression model, both adjusted for age, sex, BMI categories and smoking habits including pack-years. The results are expressed as Hazard Ratios (HR) with 95% confidence intervals (CI). Estimates with p-values<0.05 are bolded.

|  | **All** | | **Men** | | **Women** | |
| --- | --- | --- | --- | --- | --- | --- |
|  | **HR^*^** | **(95% CI)** | **HR^*^** | **(95% CI)** | **HR^*^** | **(95% CI)** |
| **All-cause mortality** |  |  |  |  |  |  |
| NLF | 1 |  | 1 |  | 1 |  |
| RSP | **1.30** | **(1.02-1.65)** | 1.32 | (0.97-1.79) | 1.19 | (0.80-1.78) |
| GOLD 1 | 1.04 | (0.80-1.36) | 1.33 | (0.95-1.87) | 0.79 | (0.51-1.22) |
| GOLD 2 | **1.32** | **(1.06-1.64)** | **1.49** | **(1.13-1.98)** | 1.06 | (0.75-1.50) |
| GOLD 3+4 | **2.84** | **(2.05-3.93)** | **2.60** | **(1.70-3.99)** | **4.10** | **(2.46-6.81)** |
| **Respiratory disease** |  |  |  |  |  |  |
| NLF | 1 |  | 1 |  | 1 |  |
| RSP | **2.69** | **(1.05-6.88)** | 2.40 | (0.74-7.79) | 2.10 | (0.46-9.50) |
| GOLD 1 | 0.75 | (0.22-2.49) | 0.56 | (0.09-3.53) | 1.23 | (0.23-6.44) |
| GOLD 2 | 2.23 | (0.96-5.15) | 1.65 | (0.49-5.47) | 2.88 | (0.77-10.73) |
| GOLD 3+4 | **12.48** | **(5.27-29.55)** | **10.05** | **(3.13-32.26)** | **15.65** | **(4.44-55.25)** |
| **Cardiovascular disease** |  |  |  |  |  |  |
| NLF | 1 |  | 1 |  | 1 |  |
| RSP | 1.11 | (0.75-1.66) | 1.04 | (0.62-1.73) | 1.38 | (0.72-2.65) |
| GOLD 1 | 1.08 | (0.72-1.62) | 1.35 | (0.82-2.23) | 0.68 | (0.32-1.41) |
| GOLD 2 | **1.60** | **(1.14-2.25)** | **1.57** | **(1.01-2.46)** | 1.59 | (0.54-4.63) |
| GOLD 3+4 | 1.54 | (0.81-2.93) | 1.59 | (0.71-3.56) | 1.60 | (0.91-2.83) |
| **Cancer** |  |  |  |  |  |  |
| NLF | 1 |  | 1 |  | 1 |  |
| RSP | 1.12 | (0.71-1.78) | 1.13 | (0.64-2.01) | 0.95 | (0.42-2.11) |
| GOLD 1 | 1.11 | (0.69-1.77) | 1.18 | (0.65-2.14) | 0.99 | (0.47-2.10) |
| GOLD 2 | 1.10 | (0.73-1.63) | 1.19 | (0.72-1.97) | 0.92 | (0.47-1.81) |
| GOLD 3+4 | 0.76 | (0.35-1.67) | 0.69 | (0.26-1.88) | 0.89 | (0.25-3.12) |
| **Dementia** |  |  |  |  |  |  |
| NLF | 1 |  | 1 |  | 1 |  |
| RSP | 1.15 | (0.58-2.30) | 1.49 | (0.57-3.90) | 0.95 | (0.32-2.78) |
| GOLD 1 | 1.22 | (0.56-2.70) | 1.48 | (0.53-4.08) | 1.01 | (0.29-3.52) |
| GOLD 2 | 0.42 | (0.17-1.06) | 0.78 | (0.27-2.31) | 0.12 | (0.01-1.06) |
| GOLD 3+4 | 0.55 | (0.11-2.73) | 0.47 | (0.05-4.19) | 1.08 | (0.10-11.37) |
| **Other** |  |  |  |  |  |  |
| NLF | 1 |  | 1 |  | 1 |  |
| RSP | 1.26 | (0.64-2.46) | 1.50 | (0.65-3.48) | 0.96 | (0.31-2.96) |
| GOLD 1 | 0.67 | (0.29-1.56) | 0.74 | (0.24-2.25) | 0.59 | (0.16-2.12) |
| GOLD 2 | 0.98 | (0.51-1.87) | 1.28 | (0.57-2.93) | 0.57 | (0.20-1.64) |
| GOLD 3+4 | 1.69 | (0.65-4.37) | 1.18 | (0.29-4.82) | 3.0 | (0.85-10.52) |
|  |  |  |  |  |  |  |
